# Myocardial fibrosis in desmin-related hypertrophic cardiomyopathy

**DOI:** 10.1186/1532-429X-12-68

**Published:** 2010-11-18

**Authors:** Yi He, Zhaoqi Zhang, Daojun Hong, Qinyi Dai, Tengyong Jiang

**Affiliations:** 1 Department of Radiology, Beijing Anzhen Hospital, Capital Medical University, No. 2, Anzhen road, Chaoyang district, Beijing, China; 2 Department of Neurology, Peking University First Hospital, Xishiku St 8#, Xicheng District, Beijing,100034, China; 3 Department of Cadiology, Beijing Anzhen Hospital, Capital Medical University, No. 2, Anzhen road, Chaoyang district, Beijing, China

## Abstract

Desmin-related myopathy (DRM) is known to cause different types of cardiomyopathy. Late gadolinium enhancement cardiovascular magnetic resonance (CMR) has been shown to identify fibrosis in ischemic and non-ischemic cardiomyopathies. We present a rare case of desmin-related hypertrophic cardiomyopathy, CMR revealed fibrosis in the lateral wall of the left ventricle. CMR is superior to conventional echocardiography for the detection of myocardial fibrosis in desmin-related cardiomyopathy, which may be useful to detect early cardiac involvement and predict the patient prognosis.

## Background

Desmin is a primary element of the intermediate filament network in skeletal, cardiac and smooth muscle cells. Desmin-related myopathy (DRM) is an autosomally inherited skeletal and cardiac muscular myopathy mainly caused by mutations in the desmin gene [1]. DRM is characterized by progressive skeletal muscle weakness, cardiomyopathy, and cardiac conduction disease. The cardiac phenotype of DRM includes cardiac arrhythmias and different types of cardiomyopathy. However, cardiac symptoms are the leading cause of death in most patients [2]. Desmin-related cardiomyopathy is usually evaluated by echocardiography through functional and morphological changes. In recent years however, cardiovascular magnetic resonance (CMR) in combination with late gadolinium enhancement (LGE) imaging has been used to differentiate normal myocardium from a variety of myocardial diseases associated with necrosis or fibrosis. We describe a case of desmin-related hypertrophic cardiomyopathy with myocardial fibrosis detected by CMR, which has not been previously reported.

## Case report

A 16 year old female presented to our cardiac department with progressive exertional dyspnea, palpitation, and skeletal muscle weakness for about two years. Two years ago she was diagnosed with hypertrophic cardiomyopathy by echocardiography. Her laboratory data showed creatine kinase (CK) 996 HU/L, brain natriuretic peptide(BNP) 2634 Hpg/ml. The electrocardiogram showed atrial fibrillation. At the referring hospital, skeletal muscle biopsies and mutation screening of the desmin gene had been performed according to the neurological workup of pathology.

Tranthoracic echocardiography showed symmetrical myocardial hypertrophy of interventricular septum and the wall of left ventricle. The wall motion was normal. Left ventricular ejection fraction (LVEF) was 66%, left ventricular (LV) diastolic (DD) and systolic (SD) diameter was 40 and 26 mm, respectively. The left atrium (LA) was dilated with diameter of 56 mm.

**Figure 1:**
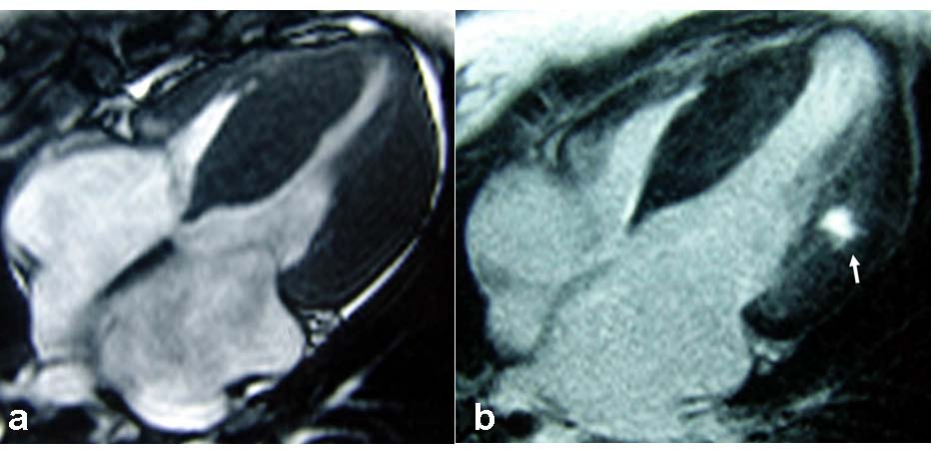
**MRI imaging of the case.** a A four-chamber view demonstrates symmetrical thickening of interventricular septum and the lateral wall of left ventricle. b A four-chamber view shows fibrosis involving the middle layer of left ventricle lateral wall (arrow).

CMR was performed with a 1.5T Magnetom Sonata (Siemens Medical Systems). Fast-gradient-echo steady-state free precession cine demonstrated symmetrical wall thicken of interventricular septum and the lateral wall of left ventricle (Figure 1a). The left atrium was enlarged. Global and regional wall motion was normal (LVEF 63%), in particular no segmental wall motion abnormality in the affected wall was observed. For myocardial tissue characterization, LGE imaging was performed with Gadolinium (Gd)-DTPA 0.2 mmol/kg BW (Magnevist, Bayer Schering Pharma, Berlin, Germany). The inversion recovery (IR) prepared 2-dimensional turboflash imaging revealed marked intramyocardial enhancement in the lateral LV wall (Figure 1b) indicating myocardial fibrosis.

**Figure 2:**
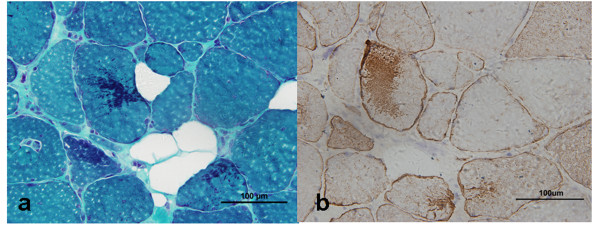
**The skeletal muscle biopsy**. Consecutive cryostat sections show some dark blue materials depositing in muscle fibers using MGT stain (a). The depositing materials are immunopositive to desmin antibody (b).

**Figure 3:**
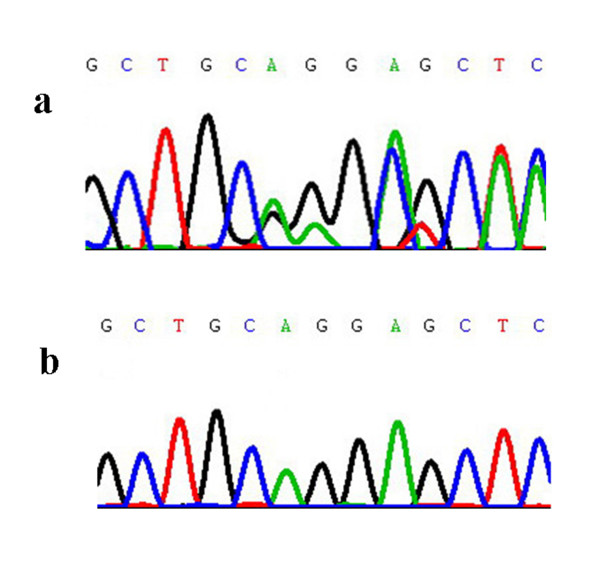
**Sequence analysis of the desmin gene**. a) The desmin gene having a c.338_339delA_G deletion mutation in exon 1. b) The control sequence of the desmin gene.

The histopathological study of skeletal muscle was in line with the pathological pictures of desminopathy. Modified Gomori staining (MGT) showed multiple dark blue materials depositing in muscle fibers (Figure 2a), which were strong immunoreactivity to desmin antibody (Figure 2b). In addition, gene screening revealed a c.338_339delA_G deletion mutation in exon 1 of the desmin gene in this patient. This mutation caused a truncated protein at codon 115 (Q113fsX115) in the helix 1A domain (Figure 3).

### Discussion

Desmin is the main intermediate filament protein expressed in skeletal, cardiac, and smooth muscle [3]. It interacts with other proteins to form a continuous cytoskeletal network that maintains a spatial relationship between the contractile apparatus and other structural elements of the cell, thus providing maintenance of cellular integrity, force transmission, and mechanochemical signaling. Primary desminopathies are caused by mutations in the desmin gene. This disease is characterized by an intracellular accumulation of insoluble protein aggregates eventually leading to cell death and replacement fibrosis [4].

The clinical manifestationsis of desminopathies are quite broad including progressive skeletal myopathy, typically presents with lower and later with upper limb muscle weakness, sometimes slowly spreading to involve truncal, neck flexor, facial and respiratory muscles [5,6]. Different types of cardiomyopathy are also seen including dilated cardiomyopathy (DCM), restrictive cardiomyopathy (RCM), hypertrophic cardiomyopathy (HCM) and arrhythmogenic right ventricular cardiomyopathy (TRVC); Cardiac arrhythmias include various degrees of atrioventricular conduction block, and supraventricular and ventricular tachyarrhythmias.

Desmin-related cardiomyopathy is usually diagnosed with echocardiography through characteristically functional and morphological changes. Cardiomyopathy has been reported in up to 50% of desmin gene mutation carriers, with DCM and RCM being the most prevalent form, but HCM has seldom been reported. In recent years, CMR has evolved as the non-invasive reference standard for accurate and highly reproducible determination of cardiac function and morphology. But the study of desmin-related cardiomyopathy with CMR is really rare. Only one study systematically used CMR imaging to evaluate myocardial involvement in desminopathies. It revealed subtle focal hypertrophic myocardial changes in two desmin-related cardiomyopathy patients which were missed by echocardiography [7]. The reason may due to the high spatial resolution and clear delineation of endocardial borders of CMR, making it more sensitive for the detection of global and focal LV pathologies and subtle change of cardiac morphology than echocardiology that sometimes affected by limited acoustic windows.

Another important finding of CMR is the detection of fibrotic intramyocardial lesions by LGE imaging. It is the only direct and noninvasive imaging technique which can find myocardial fibrosis in cardiomyopathy. The finding of myocardial fibrosis is especially noteworthy since some desmin-related cardiomyopathies with myocardial fibrosis were not associated with global or focal systolic wall motion abnormalities and therefore might be missed by wall motion studies. The extent of myocardial fibrosis has been related to patient prognosis, including worsening of LV function and adverse outcome [8].

In conclusion, CMR was able to detect myocardial fibrosis in desmin-related hypertrophic cardiomyopathy. CMR may prove useful to detect early cardiac involvement and predict the patient prognosis of desminopathies.

## Consent

Written informed consent was obtained from the patient for publication of this case report and any accompanying images. A copy of the written consent is available for review by the Editor-in-Chief of this journal.

## Competing interests

The authors declare that they have no competing interests.

## Authors' contributions

YH: Literature research, manuscript preparation and editing. ZZ: Revising the manuscript. DH: Carried out the molecular genetic and pathological studies. QD: Picture editing. TJ: Case collection.

All authors read and approved the final manuscript.

## Authors' information

YH: MD, Deptartment of Radiology, Beijing Anzhen Hospital, Capital Medical University, Beijing, 100029, China

## References

[B1] Goldfarb LG Park KY Cervenáková L Gorokhova S Lee HS Vasconcelos O Nagle JW Semino-Mora C Sivakumar K Dalakas MC Missense mutations in desmin associated with familial cardiac and skeletal myopathy Nat Genet 1998 19 402 403 10.1038/1300 9697706

[B2] Dalakas MC Park KY Semino-Mora C Lee HS Sivakumar K Goldfarb LG Desmin myopathy, a skeletal myopathy with cardiomyopathy caused by mutations in the desmin gene N Engl J Med 2000 342 770 780 10.1056/NEJM200003163421104 10717012

[B3] Lazarides E Intermediate filaments as mechanical integrators of cellular space Nature 1980 283 249 255 10.1038/283249a0 7188712

[B4] Fuchs E Cleveland DW A structural scaffolding of intermediate filaments in health and disease Science 1998 279 514 519 10.1126/science.279.5350.514 9438837

[B5] Fischer D Kley RA Strach K Meyer C Sommer T Eger K Rolfs A Meyer W Pou A Pradas J Heyer C M Grossmann A Huebner A Kress W Reimann J Schröder R Eymard B Fardeau M Udd B Goldfarb L Vorgerd M Olivé M Distinct muscle imaging patterns in myofibrillar myopathies Neurology 2008 71 758 765 10.1212/01.wnl.0000324927.28817.9b 18765652 PMC2583436

[B6] Schröder R Vrabie A Goebel HH Primary desminopathies J Cell Mol Med 2007 11 416 426 10.1111/j.1582-4934.2007.00057.x 17635637 PMC3922350

[B7] Strach K Sommer T Grohé C Meyer C Fischer D Walter MC Vorgerd M Reilich P Bär H Reimann J Reuner U Germing A Goebel HH Lochmüller H Wintersperger B Schröder R Clinical, genetic, and cardiac magnetic resonance imaging findings in primary desminopathies Neuromuscul Disord 2008 18 475 482 10.1016/j.nmd.2008.03.012 18504128

[B8] Kwon DH Smedira NG Rodriguez ER Tan C Setser R Thamilarasan M Lytle BW Lever HM Desai MY Cardiac Magnetic Resonance Detection of Myocardial Scarring in Hypertrophic Cardiomyopathy Correlation With Histopathology and Prevalence of Ventricular Tachycardia J Am Coll Cardiol 2009 54 242 249 10.1016/j.jacc.2009.04.026 19589437

